# Long non‐coding RNA‐mediated modulation of endoplasmic reticulum stress under pathological conditions

**DOI:** 10.1111/jcmm.18561

**Published:** 2024-07-27

**Authors:** Yusuf Cem Çiftçi, Yiğit Yurtsever, Bünyamin Akgül

**Affiliations:** ^1^ Noncoding RNA Laboratory, Department of Molecular Biology and Genetics Izmir Institute of Technology Izmir Turkey

**Keywords:** ATF6, disease, endoplasmic reticulum stress, IRE1, long non‐coding RNAs, PERK, unfolded protein response

## Abstract

Endoplasmic reticulum (ER) stress, which ensues from an overwhelming protein folding capacity, activates the unfolded protein response (UPR) in an effort to restore cellular homeostasis. As ER stress is associated with numerous diseases, it is highly important to delineate the molecular mechanisms governing the ER stress to gain insight into the disease pathology. Long non‐coding RNAs, transcripts with a length of over 200 nucleotides that do not code for proteins, interact with proteins and nucleic acids, fine‐tuning the UPR to restore ER homeostasis via various modes of actions. Dysregulation of specific lncRNAs is implicated in the progression of ER stress‐related diseases, presenting these molecules as promising therapeutic targets. The comprehensive analysis underscores the importance of understanding the nuanced interplay between lncRNAs and ER stress for insights into disease mechanisms. Overall, this review consolidates current knowledge, identifies research gaps and offers a roadmap for future investigations into the multifaceted roles of lncRNAs in ER stress and associated diseases to shed light on their pivotal roles in the pathogenesis of related diseases.

## 1 INTRODUCTION

The endoplasmic reticulum (ER), long underestimated, plays crucial roles in cellular function, including protein synthesis, folding, transportation and storage of calcium, lipids, glycoproteins and steroids.^1^ The ER's dynamic structure and interconnectedness with other organelles, like the Golgi apparatus and mitochondria, facilitate its multifaceted functions.^2^ Despite its significance, the ER is susceptible to stressors such as chemical insults, disruptions in cellular processes and genetic mutations, hypoxia and nutrient deprivation, threatening cell viability. Stress response mechanisms, notably involving GRP78, help mitigate these challenges. Failure in ER function can disrupt protein synthesis, underscoring its pivotal role in maintaining cellular homeostasis.^3^ Recognizing the ER's importance has led to significant advances in understanding cellular physiology and pathology. Efforts to decipher the intricate mechanisms governing ER function and stress response continue to shed light on fundamental cellular processes and offer potential avenues for therapeutic intervention in various diseases linked to ER dysfunction.

## 2 MOLECULAR MECHANISMS OF UNFOLDED PROTEIN RESPONSE

ER‐bound proteins undergo quality control prior to processing for downstream events. In contrast to DNA synthesis, protein synthesis displays a noticeable tendency for errors.^4^ Several factors can contribute to the occurrence of these errors, including incorrect pairing of cysteine residues on disulfide bonds, which hampers proper conformation.^5^, ^6^ Other potential causes include misacylation, incorrect selection of tRNA molecules or improper selection of start codons.^7^, ^8^ Even minor deviations from the typical situation might disturb the process of protein production, resulting in the buildup of unfolded or misfolded proteins within the ER.^9^ Nevertheless, in order to reduce these discomforts, cells have developed and preserved a response mechanism known as the unfolded protein response (UPR).^10^ The main goals of this mechanism include the detection of unfolded or misfolded proteins and the inhibition of their translation to sustain cellular stability. Additionally, it aimes to enhance the transcriptional activity of chaperones involved in protein synthesis^11^ and facilitate the degradation of unwanted proteins.^12^ When an organism accumulates misfolded proteins, a signalling mechanism is activated that transmits a signal across the ER membrane, ultimately leading to the overexpression of effector proteins. This upregulation subsequently results in a decrease in overall protein synthesis.^13^ Chaperones have the capacity to influence protein production as catalysts directly or indirectly. Certain chaperones display direct catalytic activity, whereas others primarily serve to maintain a folding‐competent state. These functions aim to manage the number of irreversible misfolding events, hence rendering protection to the cell.^14^ One of the most abundant ER chaperone proteins is GRP78, also referred to as binding immunoglobulin protein (BiP). The upregulation of GRP78 expression is observed in response to stress, serving as a mechanism for cellular survival.^15^ GRP78 activates glucose‐regulated protein 94 (GRP94), stimulating a conformational change that is necessary for the correct folding of proteins. In other words, the collaborative work of GRP78 and GRP94 helps minimize protein folding errors.^16^ Other well‐known ER chaperones are calnexin (CNX) and calreticulin (CRT), which, in tandem with their cochaperone ERp57, play a crucial role in overseeing partially folded glycoproteins within the ER. ERp57 specifically regulates the fate of these proteins, dictating whether they are to be released or to undergo degradation.^17^ Other types of ER chaperones include protein disulfide isomerases (PDI) and peptidyl‐prolyl cis/trans isomerases (PPIases), which act as helpers of protein folding.^18^, ^19^ The chaperones play a vital part in the maintenance of ER homeostasis, hence making a significant contribution to the longevity of cells.^20^, ^21^

**FIGURE 1:**
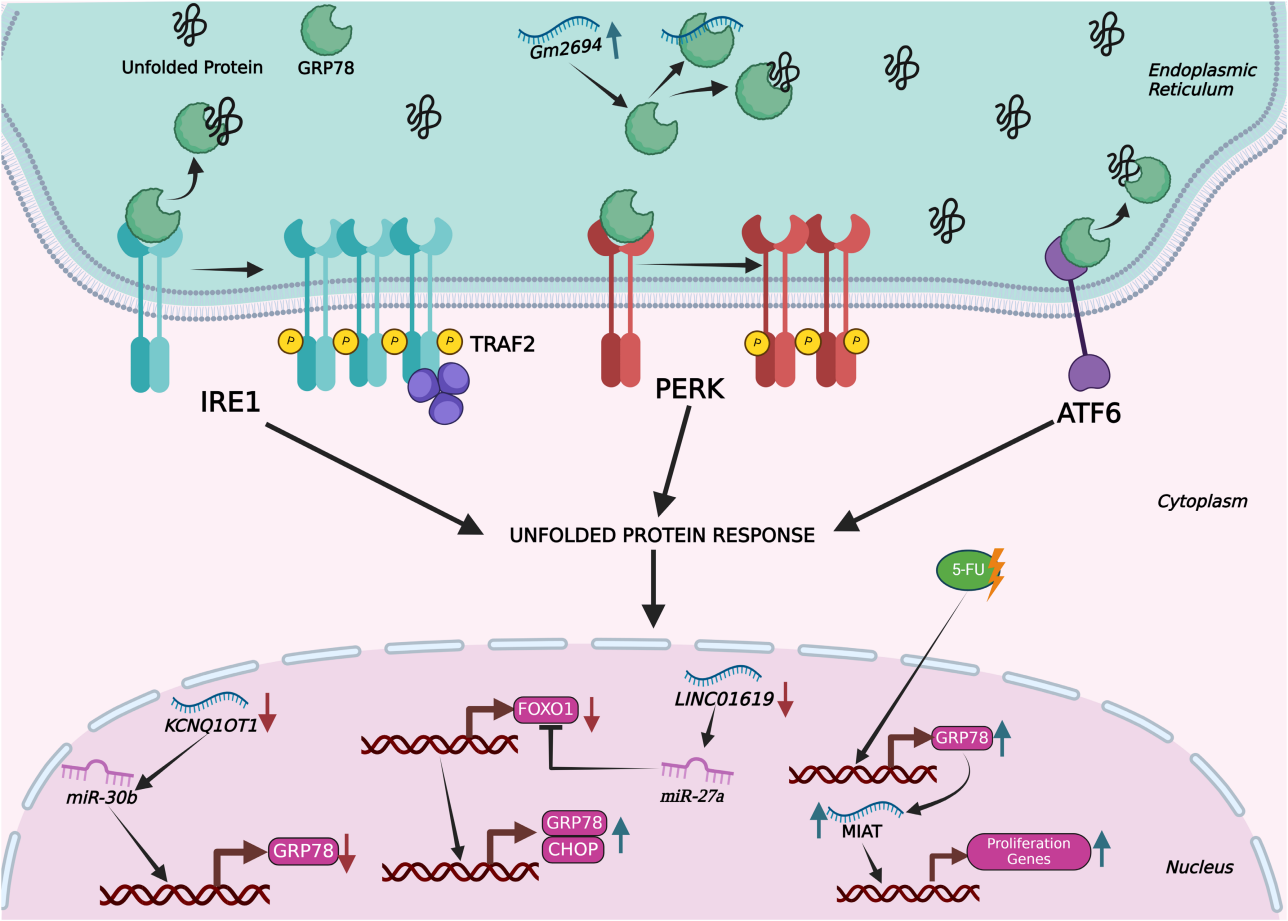
General mechanism of the unfolded protein response. UPR is activated by the accumulation of unfolded or misfolded proteins in ER, involving three primary signalling branches: IRE1, PERK and ATF6. The major ER chaperone GRP78 (BiP) regulates these sensors by binding and inhibiting them under non‐stress conditions; upon ER stress, GRP78 dissociates, allowing sensor activation. The figure also highlights non‐coding RNAs that interact with GRP78, illustrating their role in modulating the UPR.

ER chaperons also facilitate the degradation of the unfolded proteins through a process known as ER‐associated degradation (ERAD).^22^ ERAD is modulated by the ubiquitin–proteasome system (UPS), consisting of four components.^23^ The initial step involves the recognition of target proteins, mediated by a variety of chaperones and chaperone‐like lectins, including CNX and CRT.^24^, ^25^ The second step involves polyubiquitination, wherein certain proteins engage with a ubiquitin‐activating enzyme (E1). Subsequently, this complex forms a covalent bond with a ubiquitin‐conjugating enzyme (E2), and eventually, it interacts with a ubiquitin ligase (E3) to facilitate the catalytic process.^26^ The third step involves the translocation of target proteins to the cytosol, a process assisted by E3 ubiquitin ligases, which then deliver them to the Hrd complex.^27^ Subsequent to the marking and transportation of misfolded proteins into the nucleus, proteolysis degrades proteins into smaller peptides, which represents the final stage of the ERAD pathway.^12^ If these processes fail to restore the cellular homeostasis, cell death is triggered in order to eradicate the irrecoverably damaged cells.^28^ UPR is modulated by three major signalling pathways, which are conserved across the eukaryotic domain (Figure 1).^29^, ^30^

### 2.1 IRE1 signalling pathway

**FIGURE 2:**
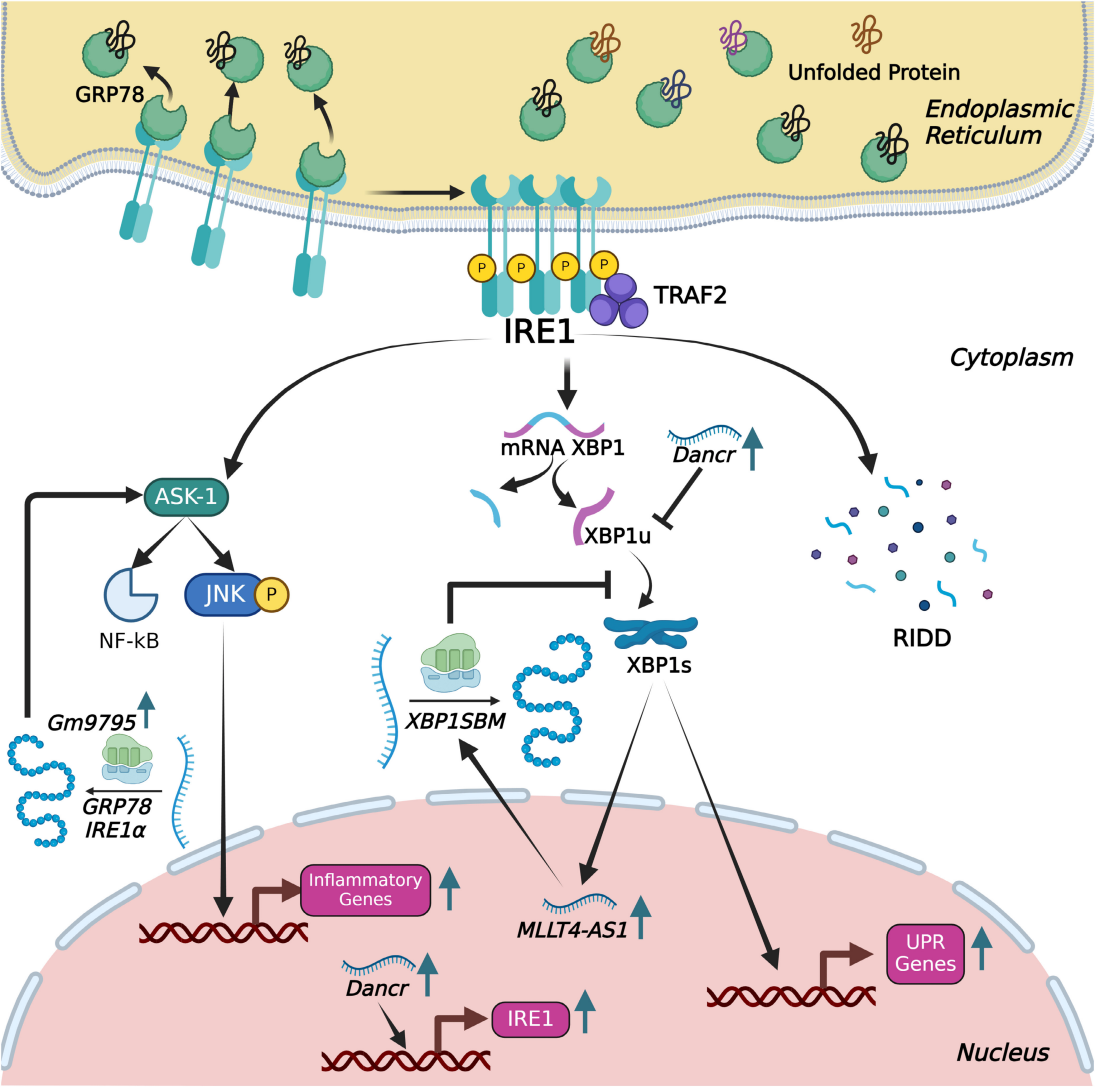
Molecular mechanism of inositol‐requiring enzyme 1 during the unfolded protein response. This figure illustrates the molecular mechanism of IRE1 during the UPR, highlighting three specific pathways. Upon ER stress, IRE1 oligomerizes and autophosphorylates, activating its endoribonuclease activity. In the XBP1 splicing pathway, IRE1 cleaves XBP1 mRNA, producing XBP1s that functions as a transcription factor to upregulate UPR genes. In the RIDD pathway, IRE1 degrades select mRNAs, reducing protein load and alleviating ER stress. The third pathway involves IRE1 interacting with the adaptor protein TRAF2, leading to the activation of ASK1 and subsequently JNK, which can promote apoptosis under prolonged ER stress. Additionally, non‐coding RNAs, including microRNAs and long non‐coding RNAs, interact with IRE1 and its targets, modulating the UPR.

The inositol‐requiring enzyme 1 (IRE1) is a well‐known and highly conserved member of the UPR.^31^ IRE1 is classified as a type ER membrane protein, containing three distinct domains: an ER luminal domain, a cytosolic kinase domain and a cytosolic endoribonuclease (RNase) domain.^32^ The luminal domain serves as a stress sensor, whereas the remaining two domains assist in the transmission of signals to relevant parts.^33^ The IRE1 signalling pathway displays distinct branches in response to ER stress (Figure 2). An increase in the amount of unfolded proteins leads to dissociation of GRP78 from IRE1. Thus, oligomerization takes place among IRE1 membrane proteins, resulting in the transmission of signals from the ER to the cytosol.^34^, ^35^ Subsequent to the stabilization of IRE1 via auto‐phosphorylation, the activated IRE1α orchestrates DNA repair mechanisms through its endonuclease domain. Activated IRE1α also modulates the splicing of X‐box binding protein 1 (XBP1) mRNA through alternative splicing to generate the functional spliced XBP1 (XBP1s) (Figure 2).^36^ XBP1 is a crucial regulator involved in the activation of target genes that are responsible for maintaining ER homeostasis.^31^, ^34^ Furthermore, IRE1α independently regulates the transformation of specific mRNA molecules encoding membrane proteins via a process known as regulated IRE1‐dependent decay (RIDD).^37^, ^38^, ^39^ Therefore, it has the ability to coordinate the components linked to ERAD to promote cellular viability.^40^ In the event of perpetuated UPR, the binding of IRE1α to tumour necrosis factor (TNF) receptor‐associated factors 2 (TRAF2) activates apoptosis signal‐regulating kinase (ASK‐1) and downstream components of C‐Jun NH2 terminal kinase (JNK) within the NF‐κB signalling pathway. Consequently, this cascade of events results in an elevated level of apoptosis and an inflammatory response.^41^, ^42^

### 2.2 ATF6 signalling pathway

**FIGURE 3:**
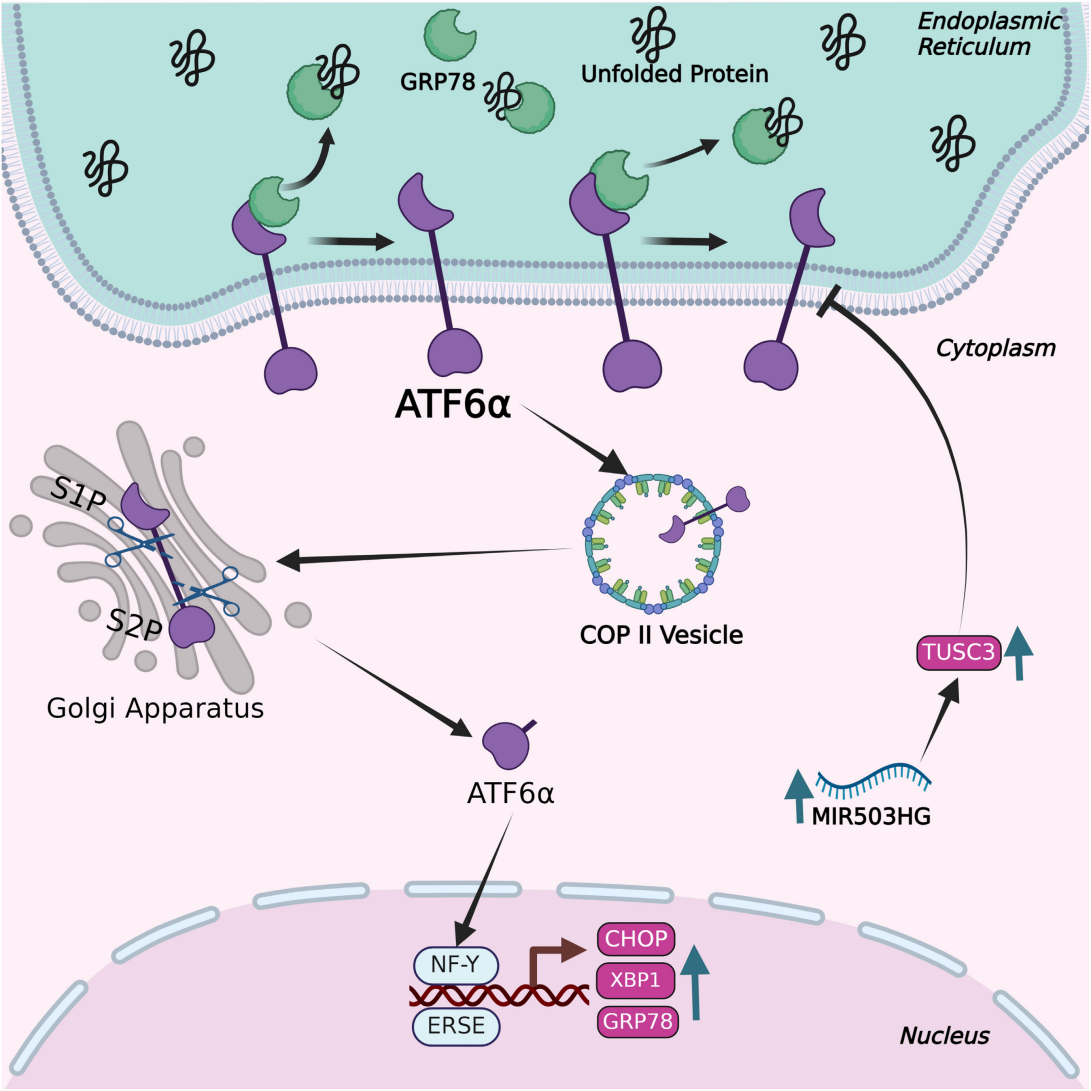
Molecular mechanism of activating transcription factor 6 during the unfolded protein response. This figure illustrates the molecular mechanism of ATF6 during the UPR, highlighting its activation process and the involvement of non‐coding RNAs. Under ER stress conditions, ATF6 translocates from the ER to the Golgi apparatus, where it is cleaved by site‐1 and site‐2 proteases. This cleavage releases the cytoplasmic domain of ATF6, which then moves to the nucleus. In the nucleus, cleaved ATF6 acts as a transcription factor, upregulating UPR target genes that enhance protein folding capacity, and other stress response elements. Additionally, non‐coding RNAs, including microRNAs and long non‐coding RNAs, interact with ATF6 and its downstream targets, modulating the UPR.

The activating transcription factor 6 (ATF6) is a transmembrane protein with a basic leucine zipper (bZIP) domain that is anchored to the ER.^43^ It is a conserved transcription factor with two homologues, ATF6α and ATF6β in mammals, that coordinates the expression of genes involved in UPRs.^44^ ATF6α displays strong transcriptional activation capabilities in response to cellular stress, while ATF6β is a comparatively weaker transcription factor.^45^ During stress induction, ATF6α undergoes translocation from the ER to the Golgi apparatus and undergoes proteolytic cleavage by site‐1 protease (S1P) and site‐2 protease (S2P) (Figure 3).^46^ The cleaved ATF6α binds to ER stress response elements (ERSE) by forming a dimer with general transcription factor NF‐Y, thereby enhancing the transcriptional activity of ER stress‐related genes such as GRP78 and XBP1.^47^, ^48^ Multiple studies have demonstrated that the lack of ATF6 leads to increased cellular vulnerability to ER stress.^49^, ^50^

### 2.3 PERK signalling pathway

**FIGURE 4:**
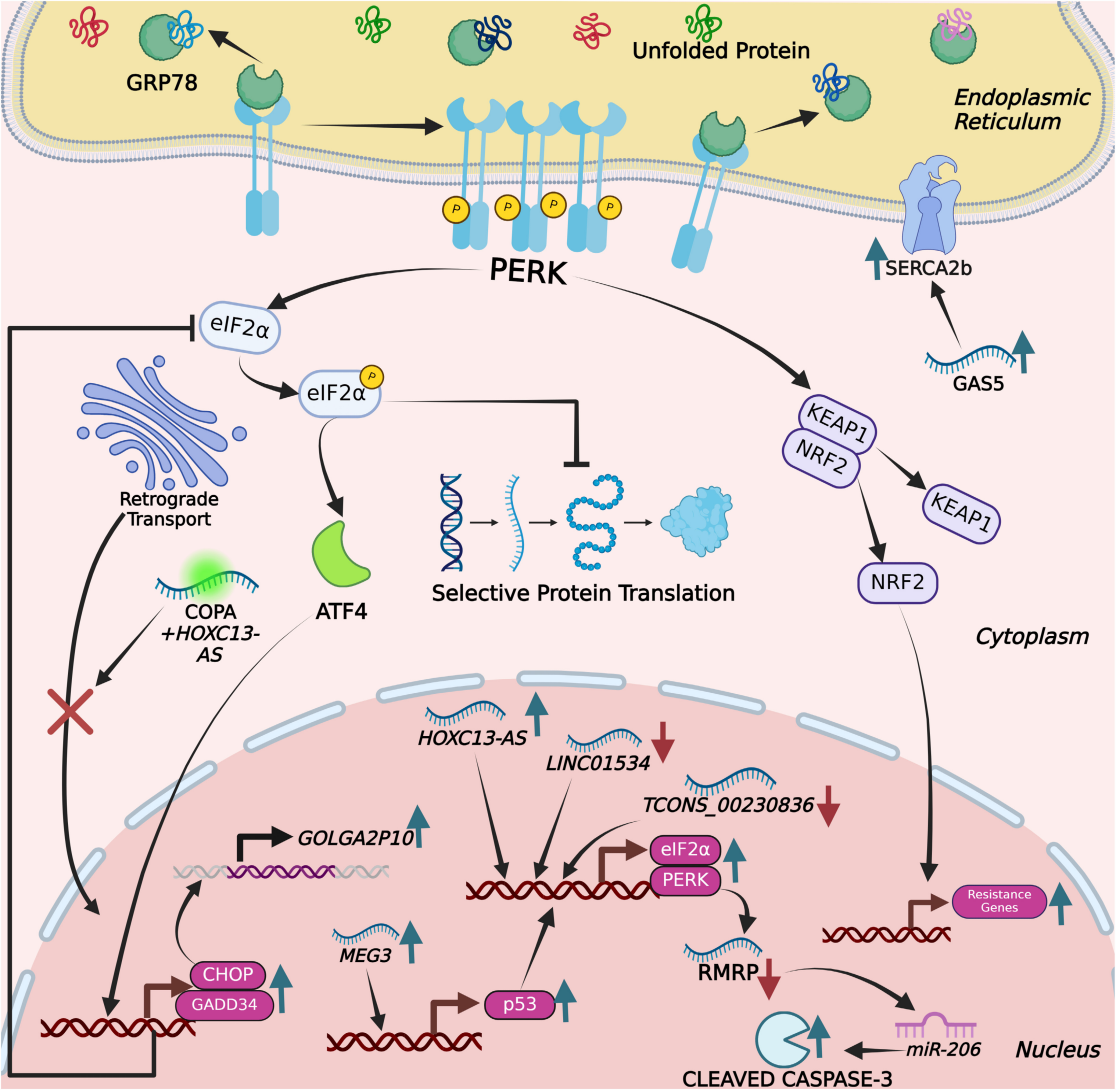
Molecular mechanism of protein kinase RNA‐activated‐like ER Kinase during the unfolded protein response. This figure illustrates the molecular mechanism of PERK during the unfolded protein response (UPR), focusing on two specific pathways. Upon ER stress, PERK autophosphorylates and phosphorylates eIF2α, reducing global protein synthesis while selectively increasing the translation of ATF4. ATF4 upregulates genes involved in antioxidant responses, amino acid metabolism and apoptosis. Additionally, PERK stabilizes and activates Nrf2 by promoting its release from Keap1, enabling Nrf2 to enter the nucleus and activate oxidative stress response genes. Non‐coding RNAs, including microRNAs and long non‐coding RNAs, interact with PERK and its targets, modulating the UPR.

Protein kinase RNA‐activated‐like ER kinase (PERK) is a member of eukaryotic initiation factor 2 (eIF2α) kinase subfamily.^51^ The activation of PERK, in the context of ER stress, requires dissociation of GRP78 from PERK, its oligomerization and phosphorylation of the α‐subunit of eIF2α, which leads to inhibition of protein translation.^52^, ^53^ However, activating transcription factor 4 (ATF4) is not prone to such translational inhibition, which, in turn, activates the integrated stress response (ISR) to support cell survival (Figure 4).^54^, ^55^ ATF4 is a transcription factor that activates several crucial genes, such as CHOP and GRP78. Subsequently, increased transcription of CHOP serves as a reliable signal of cellular death.^56^, ^57^ As part of the adaptive response, ATF4 promotes the expression of GADD34. It forms a complex with protein phosphatase 1 (PP1), which specifically de‐phosphorylates eIF2α. The de‐phosphorylation of eIF2α by the GADD34‐PP1 complex reduces the phosphorylation levels of eIF2α, thereby restoring general protein synthesis. This negative feedback mechanism serves to balance the stress response and help the cell recover by resuming normal protein synthesis once the stress is alleviated.^58^, ^59^ Upregulation of GADD34 facilitates cellular recovery from stress. Nuclear factor erythroid 2‐related factor 2 (NRF2) is another downstream protein of phosphorylated PERK, functioning as a transcription factor that modulates cellular defence mechanisms (Figure 4).^60^ The dissociation of NRF2 from KEAP1 is influenced by multiple factors, one of which is the phosphorylation of NRF2 by PERK. Under conditions of oxidative or ER stress, PERK is activated and phosphorylates NRF2. This phosphorylation event contributes to NRF2's release from KEAP1, allowing NRF2 to translocate into the nucleus where it activates the expression of antioxidant response element (ARE)‐driven genes. These genes are critical for maintaining cellular redox homeostasis and managing oxidative stress.^61^, ^62^

## 3 ER STRESS IN DISEASES

The toxicity of misfolded proteins is considerably elevated during the event of aggregation.^63^ The observed toxicity could be associated with a class of disorders known as conformational diseases or protein misfolding diseases (Table 1).^64^, ^65^ Misfolded proteins, when aggregated, exhibit heightened toxicity, contributing to conformational diseases like atherosclerosis. Atherosclerosis involves arterial narrowing due to compound accumulation such as fatty substances, cholesterol, cellular waste and calcium ions, inside the inner layer of blood vessels, activating UPR and potentially leading to cardiovascular complications.^66^, ^67^ Circulating misfolded proteins exacerbate UPR in subclinical atherosclerosis, particularly with elevated GRP78 levels.^68^ The exact mechanism linking atherosclerosis and ER stress remains unclear.^69^, ^70^ Myocardial infarction (MI) interrupts oxygen and nutrient supply, inducing ER stress and UPR. PERK pathway activation during MI downregulates cardiac ion channels, leading to arrhythmia.^71^ Ischemia–reperfusifon injury (IRI) in the heart causes calcium dysregulation, activating ER stress pathways like IRE1α/JNK.^72^

**TABLE 1:** ER stress‐associated diseases and markers.

ER stress‐associated diseases	Disease subtypes	Known ER stress markers	References
Cardiovascular diseases	Atherosclerosis	GRP78	68
	Myocardial infarction (MI)	PERK	71
	Myocardial ischaemia–reperfusion injury (IRI)	IRE1/JNK	72
Neurodegenerative diseases	Alzheimer's disease (AD)	CHOP, IRE1/XBP1, PERK/eIF2α	73, 74, 75
	Parkinson's disease (PD)	PERK	76
	Huntington's disease (HD)	PERK/eIF2α	77
	Amyotrophic lateral sclerosis (ALS)	PERK/ATF4/eIF2α	78
	Multiple sclerosis (MS)	PERK/eIF2α	79
Viral infections	Hepatitis B virus (HBV)	GRP78, PERK/eIF2α, IRE1	80
	Hepatitis C virus (HCV)	GRP78/ATF6, IRE1/XBP1	81, 82
	Human cytomegalovirus (HCMV)	ATF6/IRE1	83
	SARS‐CoV	PERK/eIF2α/ATF4/CHOP, ATF6	84, 85
Metabolic diseases	Type‐2 diabetes mellitus (T2DM)	PERK/eIF2α	86
	Lipogenesis	ATF6	87
	Non‐alcoholic fatty liver disease (NAFLD)	PERK/eIF2α	88
	Non‐alcoholic steatohepatitis (NASH)	PERK/eIF2α, IRE1/JNK/NF‐kB	89
	Diabetic nephropathy (DN)	ATF6/CHOP	90
Cancer	Colorectal cancer (CRC)	PERK/eIF2α/ATF4/CHOP, IRE1/XBP1	91, 92
	Breast cancer (BC)	PERK/ATF4, GRP78/XBP1	93
	Triple‐negative breast cancer (TNBC)	PERK/IRE1/CHOP	94
	Hepatocellular carcinoma (HCC)	IRE1/XBP1 PERK/BCL‐2	95, 96
	Gastric cancer (GC)	PERK/eIF2α/CHOP, PERK/ATF6	97, 98
Ophthalmic diseases	Diabetic retinopathy (DR)	ATF6	99
	Retinitis pigmentosa (RP)	IRE1/XBP1s, ATF6, PERK/ATF4	100, 101

Neurodegenerative diseases, such as Alzheimer's disease (AD), Parkinson's disease (PD), Huntington's disease (HD), amyotrophic lateral sclerosis (ALS) and multiple sclerosis (MS) are characterized by the accumulation of misfolded proteins, leading to neuronal damage and cell death. This accumulation triggers ER stress responses, contributing to the pathogenesis of these debilitating disorders.^102^ In AD, the hallmark features include the accumulation of amyloid‐beta (Aβ) plaques and tau tangles in the brain, leading to synaptic dysfunction and neuronal loss. CHOP pathway, a key mediator of ER stress‐induced apoptosis, has been implicated in AD pathogenesis.^73^ Additionally, dysregulation of XBP‐1 and eIF2α, downstream effectors of the UPR, further contributes to neuronal dysfunction in AD.^103^ Similarly, PD is characterized by the progressive loss of dopaminergic neurons in the substantia nigra, leading to motor impairment. The accumulation of misfolded α‐synuclein aggregates, known as Lewy bodies, contributes to ER stress and UPR activation.^104^ PERK, a key initiator of the UPR, has been detected in the brains of PD patients, indicating ER stress involvement in disease pathogenesis.^76^ HD is a hereditary neurodegenerative disorder caused by an expanded CAG repeat in the huntingtin (HTT) gene, leading to the production of mutant huntingtin protein (mHTT). The aggregation of mHTT induces ER stress and activates the UPR, particularly through the PERK/eIF2α pathway.^77^ Inhibition of PERK has shown promise in mitigating disease progression in HD models, highlighting the therapeutic potential of targeting ER stress pathways. ALS is characterized by the progressive degeneration of motor neurons, leading to muscle weakness and paralysis. Misfolded proteins, including superoxide dismutase 1 (SOD1) and TAR DNA‐binding protein 43 (TDP‐43), accumulate in affected neurons, triggering ER stress and UPR activation. The PERK/eIF2α pathway plays a crucial role in modulating neuronal survival in ALS, while its dysregulation may contribute to neuronal loss.^105^ MS is an autoimmune disorder characterized by inflammation and demyelination in the central nervous system. ER stress and UPR activation have been implicated in MS pathogenesis, with the PERK/eIF2α pathway influencing cell survival in response to stress.^106^ However, the role of other UPR components, such as ATF4, in MS pathology requires further investigation.

Viral infections represent a different type of disease associated with ER stress, which arises from the production of foreign genetic material, leading to the buildup of poorly folded proteins. Hepatitis B virus (HBV) can be considered one of the common viruses where the ER stress agents are activated via HBx proteins' direct involvement with GRP78 resulting in inhibition of the PERK pathway and activation of IRE1α.^80^ Hepatitis C Virus (HCV) is also associated with ER stress.^107^ Two different studies have reported the cleavage of ATF6 and the induction of GRP78 in HCV.^82^, ^108^ Other studies showed the suppression of the IRE1α/XBP1 pathway which is tightly involved with the UPR.^81^ Human cytomegalovirus (HCMV) infection triggers the unfolded UPR but selectively modulates its three branches to favour viral replication. Specifically, HCMV limits eIF2α phosphorylation to avoid translation attenuation, suppresses ATF6 activation while still inducing chaperone genes and activates the IRE‐1 pathway but inhibits transcription of certain degradation factors.^83^ It is proposed that the PERK and IRE1α branches of the UPR are detrimental to viral infections while the ATF6 pathway is beneficial.^108^, ^109^ The viral infection that the world has become recently acquainted with, namely, SARS‐CoV, is also tightly associated with ER stress via the PERK/eIF2α/ATF4/CHOP axis by 3a proteins.^84^, ^110^ There are also reports that SARS‐CoV infection involves the ATF6 axis of the UPR via 8ab proteins.^85^

The PERK pathway has a strong association with a loss of beta‐cell function and the development of insulin resistance, both of which are key factors in the pathogenesis of diabetes.^111^ Inhibition of ATF6α in mice triggers glucose intolerance due to beta‐cell loss of function, which could lead to diabetes. Especially in the case of Type‐2 Diabetes Mellitus (T2DM), the PERK/eIF2α pathway could be downregulated via Gymnemic acid, which could help alleviate ER stress.^86^ Since ER regulates lipid and protein synthesis, any fluctuation at the glucose level could trigger ER stress and UPR branches.^112^ For example, ATF6 could modulate lipogenesis via binding sterol regulatory element binding proteins (SREBPs) and decreasing SREBP levels, resulting in an increase in GRP78 levels.^87^ Another important and common metabolic disease, non‐alcoholic fatty liver disease (NAFLD) and non‐alcoholic steatohepatitis (NASH), could affect liver homeostasis through the accumulation of fat also known as hepatic steatosis.^113^ These diseases are closely linked to each other via different downstream effectors of UPR. For example, both NAFLD and NASH involves activation of the PERK/eIF2α pathway while downstream of this pathway, the ATF4/CHOP/GADD34 branch, remains inactive. Additionally, NASH also seems to be associated with the activation of the IRE1/JNK/NF‐kB pathway.^114^ Another interesting study reports that the ATF6 pathway of the UPR is inhibited and the IRE1 pathway is activated resulting in sXBP1 activation under normal conditions while the lack of insulin promotes the ATF6‐mediated activation of CHOP that could lead to cell death in diabetic nephropathy (DN).^90^

Cancer cells display an elevated interest in ER stress because of the particular environment they inhabit, characterized by increased glucose deprivation and hypoxia.^115^ These conditions require enhanced ER activity, consequently leading to the development of ER stress. UPR is triggered by hypoxia, with the activation of the ISR signalling to promote cellular survival in conditions of severe hypoxia, hence playing a crucial role in the progression of malignancy.^116^ Multiple studies have demonstrated that suppression of specific ER stress mediators, such as PERK and GRP78, leads to a reduction in cancer progression and vulnerability to malignancies.^117^ Furthermore, ER stress promotes the activation of some cellular drug resistance mechanisms that could impact the growth of tumour cells.^118^ In colorectal cancer (CRC), the PERK/eIF2α/ATF4/CHOP pathway is activated via ciclopirox (CPX), which triggers cell death.^91^ ER stress‐associated cell death could be triggered by the IRE1α/XBP‐1 pathway in addition to the PERK/eIF2α pathway. In breast cancer (BC), the PERK/ATF4 pathway could be inhibited to mitigate radioresistance, sensitizing tumours to radiotherapy.^93^ Other components of the UPR may be affected in BC. For example, GRP78 and XBP1 are differentially expressed in response to the stimuli stemming from the tumour microenvironment. In turn, overexpression of specific ER stress genes enhances cell survival by facilitating resistance to apoptosis.^119^ Triple‐negative breast cancer (TNBC) represents the least treatable type of breast cancer. Ilamycin E promotes ER stress‐induced apoptosis resulting in activation of CHOP and inhibition of Bcl‐2.^94^ The IRE1α‐XBP1 pathway promotes cell proliferation and progression of hepatocellular carcinoma (HCC) with other signalling pathways involved.^95^ Insulin resistance promotes UPR by activating the PERK/Bcl‐2 pathway and inhibiting caspase‐3‐dependent apoptosis pathway, resulting in the survival of tumour cells in liver cancer cells.^96^ In gastric cancer (GC), activation of the PERK/eIF2α/CHOP axis could lead to apoptosis in gastric epithelial cells.^97^ Interestingly, ATF6 and PERK pathways could protect the cells from apigenin‐induced apoptosis.^98^ Thus, targeting ER stress represents itself as a potential therapeutic approach in GC.

Recently, different studies have focused on ophthalmic diseases and their relationship with ER stress and UPR branches.^64^ In one of the ophthalmic diseases, diabetic retinopathy (DR), diabetes damage blood vessels in the retina causing blindness in people with diabetes. Interestingly, transcription factor 7 like 2 (TCF7L2), a susceptibility gene for DR, contributes to the pathogenesis of DR by activating ER stress through ATF6 that results in vein endothelial cell permeability.^99^ Retinitis pigmentosa (RP) is another ophthalmic disease which is a common form of hereditary blindness characterized by the degeneration of photoreceptors. RP pathogenesis is associated with ER stress.^64^ Rhodopsin is required for low light visualization where the inhibition of ATF6 results in hyperactivation of IRE1/XBP1s consequently leading to ERAD for the alleviating of misfolded rhodopsin. Thus, it is crystal clear that ATF6 is crucial for efficient rhodopsin clearance and could be targeted for a therapeutic approach in RP.^100^ There are also reports that photoreceptor cell death may be modulated by ER stress. For example, the PERK pathway serves a protective role in preventing photoreceptor cellular death via ınhibition of IRE1‐induced autophagy.^101^ Furthermore, ATF4 overexpression triggers photoreceptor cell death.

## 4 LONG NON‐CODING RNAs

The completion of genome projects followed by genome annotations has revealed that less than 2% of the human genome is translated into proteins. The rest of the genome is not useless as the majority of the genome is transcribed into non‐coding transcripts in at least one cell type under at least one cellular condition.^120^ Non‐coding RNAs (ncRNAs) are defined as transcripts without protein‐coding capacity. Typically, three criteria are employed to assess whether an ncRNA possesses a biological function: (1) conservation; (2) the presence of a disease‐linked single nucleotide polymorphism (SNP) and (3) processing, such as capping, splicing or polyadenylation.^121^ Compared with mRNAs, ncRNAs show lower expression levels, although they play a crucial role in maintaining cell viability and homeostasis.^122^, ^123^ ncRNAs could be housekeeping or regulatory based on their function.^124^ The best known examples of housekeeping ncRNAs are transfer RNAs (tRNAs)^125^ and ribosomal RNAs (rRNAs).^126^ Regulatory ncRNAs are further classified into small and long ncRNAs based on their size. Several examples of functional small ncRNAs include microRNAs (miRNAs)^127^ small interfering RNAs (siRNAs), and PIWI‐associated RNAs (piRNAs).^128^ Long non‐coding RNAs (lncRNAs), which are longer than 200 nucleotides in size, can exist in either linear or circular forms.^129^ lncRNAs are classified into five distinct categories, namely bidirectional, intergenic, intronic, sense and antisense, based on their genomic locations. Bidirectional lncRNAs, similar to a protein‐coding gene, are transcribed from the same promoter but in the opposite direction, while long intergenic RNAs (lincRNAs) are located between two protein‐coding genes. Protein‐coding gene introns serve as the primary source of intronic lncRNAs, which may be produced in a transcription‐dependent or independent manner. Sense lncRNAs are transcribed from the sense strand of the protein‐coding genes, while antisense lncRNAs are transcribed from the complementary strand of the protein‐coding genes.^130^

The majority of lncRNAs is predominantly localized within the nucleus and play a crucial role in modulating epigenetic or transcriptional processes by serving as signal, decoy, guide or scaffold molecules.^121^, ^131^ As signalling molecules, lncRNAs can modulate gene expression by functioning as a coactivator.^132^ Guide lncRNAs direct different molecules, such as transcription factors, chromatin remodelers or chromatin modifying enzymes, to their site of function. Additionally, they act as dynamic scaffolds, providing an architectural platform for enzymatic complexes and molecular decoys to prevent any type of interaction resulting in transcription or translation.^133^ In the nucleus, lncRNAs play a crucial role in transcriptional regulation through transcriptional interference,^134^ chromatin remodelling^135^ and splicing.^136^ A fraction of lncRNAs may be localized in the cytoplasm where they modulate the fate of mRNAs post‐transcriptionally.^137^ Interactions between lncRNAs and RNA‐binding proteins (RBPs) may dictate the cytoplasmic fate of mRNAs by altering their stability, translation efficiency or localization. Additionally, lncRNAs may bind to their target RNAs, such as miRNAs or mRNAs, and could regulate gene expression by serving as a competing endogenous RNA.^138^ Ribosomal profiling has demonstrated that a portion of cytoplasmically localized lncRNAs may be translated into micropeptides.^139^ Based on this translation potential, polysomally associated lncRNAs are re‐defined as long non‐canonical RNAs.

### 4.1 Roles of long non‐coding RNAs in ER stress

Numerous studies have demonstrated that a diverse range of lncRNAs play a significant role in the regulation of ER stress pathways in different diseases, either directly or indirectly. In this review, we will specifically focus on studies that report a direct link between lncRNAs and ER stress (Table 2). There are several reports that link ER stress to pathological diseases in which lncRNAs ameliorate or aggravate disease progression. For example, lncRNA Gm2694, which is overexpressed in chronic social defeat stress (CSDS), directly interacts with GRP78, hence limiting its functionality in maintaining ER homeostasis. Congruently, the Gm2694‐GRP78 complex inhibits the translocation of AMPA receptors to the postsynaptic membrane, hence leading to CSDS through the activation of UPR.^140^ In diabetic nephropathy patients, lncRNA 01619 is downregulated, de‐repressing miR‐27a‐mediated suppression of Forkhead box protein O1 (FOXO1). Consequently, lncRNA 01619 downregulation triggers ER stress by upregulating the expression of GRP78 and CHOP.^141^ lncRNA KCNQ1OT1 is expressed at elevated levels in individuals suffering from cerebral ischemia–reperfusion injury. Upregulation of lncRNA KCNQ1OT1 causes reduction in the expression of microRNA‐30b, which subsequently leads to a rise in GRP78 (Figure 1). Consequently, the increased level of GRP78 induces an ongoing state of cellular stress, potentially resulting in apoptosis associated with ER stress.^142^ In ARPE‐19 cells, high‐glucose treatment results in a decrease of lncRNA GAS5 and an increase in ER stress, suggesting a link between ER stress and diabetic retinopathy^143^. Moreover, overexpression of lncRNA GAS5 increases sarco/endoplasmic reticulum Ca^2+^‐ATPase 2b also known as SERCA2b protein level while decreasing the high glucose‐induced ER stress levels via inhibition of PERK/eIF2a/ATF4/CHOP pathway (Figure 4). The function of SERCA2b channel proteins is crucial for storage and transportation of calcium ions. These observations suggest that lncRNA GAS5, could reduce the high glucose‐induced ER stress through SERCA2b channel proteins in ARPE‐19 cells. ^143^ ER stress‐induced inflammation has major significance in the pathogenesis of several disorders, including non‐alcoholic steatohepatitis (NASH), a critical stage in the progression of non‐alcoholic fatty liver (NAFL). lncRNA Gm9795 promotes the activation of IRE1α and GRP78 in in vitro models of NASH, leading to the induction of ER stress (Figure 2). This ER stress, consequently, activates the NF‐κB/JNK pathway and inflammatory markers.^144^ Inhibition of lncRNA MEG3 leads to attenuation of ER stress through downregulation of PERK/eIF2α/CHOP and the caspase 12 pathway by directly targeting p53 following myocardial infarction (Figure 4). Consequently, p53‐mediated downregulation causes a decrease in the production of the pro‐apoptotic protein Bax and an increase in the expression of the anti‐apoptotic protein Bcl‐2, thereby preventing apoptosis and providing cardioprotection.^145^

**TABLE 2:** Unfolded protein response (UPR) pathways and their specific long non‐coding RNAs (lncRNA) are shown with lncRNA‐specific targets.

Pathway	LncRNA	Targets	Cell lines	Associated disease	References
GRP78/BiP	GM2694	GRP78	C57BL/6J mice, CD‐1 mice	Chronic social defeat stress (CSDS)	140
	MIAT	GRP78	293 T, MDA‐MB‐468, and MCF‐7 cell lines	Breast cancer	146
	LINC01619	miR‐27a/FOXO1/CHOP, GRP78	immortalized murine podocyte	Diabetic nephropathy (DN)	141
	KCNQ1OT1	miR‐30b/GRP78	P12	Cerebral ischaemia–reperfusion Injury (CIRI)	142
PERK	GOLGA2P10	CHOP	MHCC‐97H, QGY‐7703, SK‐HEP‐1, 293 T	Hepatocellular carcinoma	147
	MEG3	p53	MOUSE C57/BL6J Cells	Myocardial infarction (MI)	86
	RMRP	PERK/miRNA‐206b/BCL2/Caspase3	Huh 7, HLE	Hepatocellular carcinoma	148
	LINC01534	PERK/eIF2α	HCT116, RKO	Colorectal cancer (CRC)	149
	HOXC13‐AS	COPA	Human adult epidermal keratinocytes	Chronic nonhealing wounds	150
	TCONS_00230836	PERK/eIF2α	β‐TC6 cells	Type 2 diabetes mellitus (T2DM)	151
	GAS5	SERCA2b/CHOP	ARPE‐19 cell line/ HepG2 cells	Diabetic retinopathy (DR)/hepatocellular carcinoma	143
	MLLT4‐AS1	XBP1SM	Human TNBC Tissues	Triple‐negative breast cancer (TNBC).	99
IRE1	Dancr	miR‐6324/XBP1u	H9C2	Myocardial ischaemia/reperfusion injury, myocardial infarction (MI)	152
	GM9795	IRE1/NF‐kB	AML 12 Cells	Non‐alcoholic steatohepatitis	144
ATF6	MIR503HG	miR‐224‐5p/TUSC3/ATF6	SGC7901, BGC‐823, GES‐1	Gastric cancer	153

Cancer cells are notorious for optimizing survival under the conditions characterized by increased glucose deprivation and hypoxia. These challenging conditions mandate increased ER activity and ER stress as a result. Thus, there are several studies that report the crucial role of lncRNAs in modulating the interplay between cancer and ER stress. For example, lncRNA 01534 is crucial in maintaining the cancer stemness of colorectal cancer (CRC) cells, while simultaneously inhibiting the ER stress response. Inhibition of lncRNA 01534 is associated with an increased level of PERK and phosphorylated eIF2α, which could contribute to the induction of apoptotic cell death. Therefore, it might be stated that lncRNA 01534 is likely to be involved in regulating the ER stress response in CRC cells.^149^ lncRNA MIR503HG and a signalling cascade composed of its downstream targets miR‐224‐5p and tumour suppressor candidate 3 (TUSC3) suppress cancer development by modulating ATF6 in gastric cancer.^153^ In GC patients, lncRNA MIR503HG and TUSC3 mRNA were downregulated while miR‐224‐5p was upregulated. lncRNA MIR503HG upregulation inhibits cell proliferation in PERK‐IRE1 deficient GC cells but not ATF6 deficient GC cells. These observations are taken to suggest that lncRNA MIR503HG functions as a sponge for miR‐224‐5p, which results in upregulation of TUSC3, leading to the suppression of ATF6 branch of UPR and GC development.^153^ Breast cancer (BC) cells develop resistance to 5‐fluorouracil (5‐FU), an anticancer drug, through the activation of ER stress. 5‐FU‐mediated ER stress involves upregulation of GRP78, which subsequently leads to the overexpression of lncRNA myocardial infarction‐associated transcript (MIAT) and other proteins (Figure 1). These molecular changes collectively contribute to the development of resistance to 5‐FU in BC cells.^146^ lncRNA MLLT4‐AS1 is upregulated in triple‐negative breast cancer (TNBC), which is a very aggressive subtype of BC. Glutamine deprivation promotes the activation of the IRE1α‐XBP1 pathway, leading to the upregulation of lncRNA MLLT4‐AS1 by XBP1 (Figure 2).^154^ This activation also promotes the synthesis of a micropeptide, XBP1SBM, which suppresses nonfunctional unspliced XBP1 mRNA (XBP1u) and functional spliced XBP1 mRNA (XBP1s). The presence of XBP1s is essential for the UPR process in BC. In hepatocellular carcinoma cells, PERK as a key molecule in modulating ER stress downregulates the expression of the RNA component of mitochondrial RNA processing endoribonuclease (RMRP), which is an lncRNA.^148^ RMRP downregulation increases miR‐206 and activates apoptosis by inhibiting the expression of the anti‐apoptotic protein Bcl‐2 (Figure 4).^148^

ER stress inducers tunicamycin and thapsigargin may also target certain lncRNAs to modulate the ER stress response. For example, lncRNA GOLGA2P10, which is activated by tunicamycin and thapsigargin, prevents ER stress‐mediated apoptosis by upregulating the expression of B‐cell lymphoma‐extra‐large (Bcl‐xL), an essential anti‐apoptotic protein. Additionally, lncRNA GOLGA2P10 enhances the phosphorylation of the BCL2‐associated agonist of cell death (BAD), a pro‐apoptotic protein, through the PERK/ATF4/CHOP pathway.^147^ Dancr lncRNA is suppressed by tunicamycin in cardiomyocytes, leading to apoptosis. However, Dancr overexpression activates the IRE1α/Xbp1 branch by downregulating Xbp1u and upregulating p‐IRE1α and Xbp1s. This activation of the UPR pathway protects cardiomyocytes against ER stress by sponging miR‐6324, therefore promoting ER homeostasis and cell survival.^152^

lncRNAs modulate ER stress response in metabolism and regeneration as well. For example, upregulation of lncRNA HOXC13‐AS can disrupt transportation from the Golgi apparatus to the ER by interfering with the coat complex subunit alpha (COPA) during wound healing in keratinocytes. Ultimately, these disturbances lead to ER stress through the activation of PERK, eIF2α ATF4, GRP78 and CHOP proteins (Figure 4). While it has been demonstrated that the activation of PERK may not be necessary for the pro‐differentiation function of HOXC13‐AS, the phosphorylation of eIF2α is indeed critical during regenerative processes in keratinocytes.^150^ In steric acid‐induced β‐TC6 cells, the inhibition of lncRNA TCONS_00230836 alleviates the ER stress specifically via PERK‐eIF2a pathway while the levels of ATF6 and IRE1 remaining unchanged the same under steric acid‐induced ER stress conditions (Figure 4).^151^

## 5 CONCLUDING REMARKS

Dysregulation of ER stress and its interplay with lncRNAs is an area of burgeoning research with profound implications for understanding and potentially treating various diseases. ER stress occurs when the folding capacity of ER is overwhelmed, leading to activation of UPR. While the UPR aims to restore ER homeostasis, dysregulation can contribute to disease pathogenesis. lncRNAs have emerged as critical regulators of ER stress responses, functioning as scaffolds, decoys or guides to fine‐tune the UPR by interacting with proteins and nucleic acids.

Specific lncRNAs have been implicated in the pathogenesis of ER stress‐related diseases, suggesting their potential as therapeutic targets. Understanding the molecular mechanisms of lncRNA‐mediated modulation of ER stress could lead to targeted interventions tailored to individual genetic and molecular profiles, minimizing side effects and improving outcomes. Monitoring lncRNA levels in bodily fluids or tissues could enable early disease detection, prognosis assessment and treatment response tracking, facilitating timely interventions and enhancing patient outcomes. Advancements in technologies like high‐throughput sequencing, single‐cell analysis and CRISPR‐based genome editing enhance researchers' ability to study lncRNA roles in ER stress pathways. Integrating lncRNA research with other omics data provides a comprehensive understanding of complex regulatory networks in ER stress‐related diseases, identifying new therapeutic targets.

The review highlights a limited number of lncRNAs associated with the ATF6 pathway compared with the more extensively studied PERK and IRE1 pathways in ER stress‐related diseases (Table 3). This discrepancy may stem from the broader regulatory interactions of PERK and IRE1 pathways in diverse cellular processes beyond ER stress, unlike the primarily ER‐focused role of the ATF6 pathway. However, the importance of ATF6 in various pathological conditions is increasingly recognized, warranting further exploration of its associated lncRNAs and potential therapeutic targets. Antisense lncRNAs, implicated in gene expression regulation during ER stress, are more prominent compared with other lncRNA biotypes (Table 3). Their role in modulating gene expression through RNA–RNA duplex formation with sense transcripts suggests a specific mechanism for regulating UPR or ER stress response genes. The apparent scarcity of other lncRNA biotypes may reflect the current state of research, indicating ongoing exploration of their roles in ER stress and related processes.

**TABLE 3:** Types of long non‐coding RNAs (lncRNA) related to ER stress pathways, PERK‐ATF6‐IRE1‐GRP78, are shown.

TYPES OF LncRNAs	PERK	ER STRESS	PATHWAYS	GRP78
		ATF6	IRE1	
Sense				Linc01619
Antisense	MEG3			KCNQO1T1
	HOXC13‐AS			
	LINC01534			
	GAS5			
Intergenic	TCONS_00230836	MIR503HG	Dancr	MIAT
Intronic				
Bidirectional	RMRP		MLLT4‐AS1	

In conclusion, the intricate involvement of lncRNAs in ER stress regulation underscores their significance in disease pathogenesis. Elucidating lncRNA roles in ER stress‐related pathways offers insights into disease mechanisms and potential therapeutic targets, paving the way for targeted therapeutic strategies and biomarker identification in various disorders.

## AUTHOR CONTRIBUTIONS

**Yusuf Cem Çiftçi:** Conceptualization (supporting); data curation (equal); supervision (supporting); writing – original draft (equal); writing – review and editing (equal). **Yiğit Yurtsever:** Data curation (equal); writing – original draft (equal); writing – review and editing (equal). **Bünyamin Akgül:** Conceptualization (lead); funding acquisition (lead); supervision (lead); writing – original draft (supporting); writing – review and editing (equal).

## CONFLICT OF INTEREST STATEMENT

The authors confirm that there are no conflicts of interest.

## Data Availability

Data sharing not applicable to this article as no datasets were generated or analysed during the current study.
